# Genital Prolapse Causing Urinary Obstruction and Hydronephrosis in a Neonate: A Case and Review of the Literature 

**Published:** 2012-07-01

**Authors:** Gina Lockwood, Charles Durkee, Travis Groth

**Affiliations:** Departments of Urology, Medical College of Wisconsin, 9200 West Wisconsin Avenue Milwaukee, WI 53226 and Children’s Hospital of Wisconsin PO Box 1997 Milwaukee, WI 53201.

**Keywords:** Neonatal genital prolapse, obstructive uropathya, myelomeningocele

## Abstract

Neonatal genital prolapse is a rare condition seen early in life, often in conjunction with spinal cord anomalies. We present a case of a 38-week gestational age female in whom urinary obstruction and bilateral hydronephrosis resulted from genital prolapse. We suggest that although a serious urologic outcome can potentially result from this condition, cure for both can be achieved swiftly and without major complications.

## CASE REPORT

A 38-week gestational age female was born to a gravida 2, para 2 mother following in-utero diagnosis of myelomeningocele at seven months. After birth, the patient was diagnosed with lumbar myelomeningocele, hydrocephalus, right hip subluxation, left hip dislocation with acetabular dysplasia, bilateral metatarsus adductus, and vaginal and uterine prolapse. Renal ultrasound obtained on the first day of life revealed normal upper tracts and no hydronephrosis. The prolapse was initially treated conservatively with moistened gauze, and her myelomeningocele was surgically closed. Renal ultrasound repeated at age ten days revealed new bilateral grade IV hydronephrosis with moderate bladder distention. Creatinine was within normal limits. She was started on intermittent straight catheterization for bladder management. However, it is unclear if this was performed appropriately as parent compliance was a recurrent issue with this patient. She underwent ventriculoperitoneal shunt placement on day 20 of life. Repeat ultrasound at the age of 22 days showed progression of her hydronephrosis (Fig.1).

**Figure:**
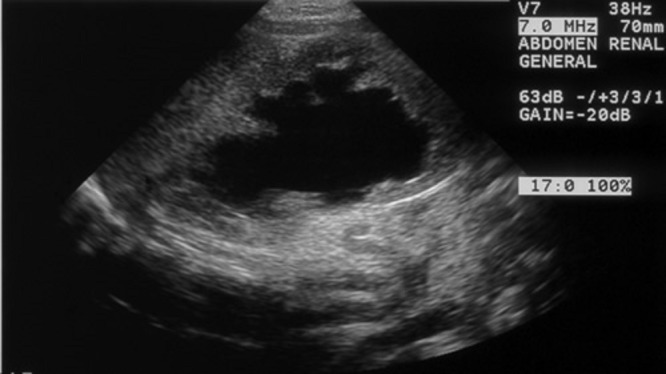
Figure 1: Neonatal renal ultrasound performed prior to operative intervention for prolapse, showing severe left hydronephrosis. Hydronephrosis on the right was comparable in severity.

At 29 days, the patient was taken to the operating room with the goals to define her anatomy, evaluate and reduce her genital prolapse, and potentially provide a means to relieve her obstruction and hydronephrosis. After a single digit was used to reduce the prolapse, a 12 F urethral sound and a 10 F cystoscope were easily passed. Cystoscopy and bilateral retrograde pyelograms revealed bilateral hydroureteronephrosis to the level of the ureterovesical junction, left more severe than right. Given the high degree of hydronephrosis on the left, there was concern for primary obstructive megaureter, and it was unclear at the time if the obstruction was simply due to her prolapse or megaureter. Therefore a 7x12 double-J ureteral stent was placed on the left. The prolapse was then reduced and a vaginal pessary was placed. The pessary was created from a medium size vaginal dilator with 1/3 of the lip excised. This was secured into place with six 5-0 prolene stitches. The pessary was removed after one month during which every four-hour intermittent catheterization had been continued, and bulky pads had been applied to her diaper to apply gentle pressure to the vaginal dilator. At the time of pessary removal, her bilateral hydronephrosis and prolapse had completely resolved with no change in her bladder management, indicating that the prolapse had been the cause of her obstruction. Her ureteral stent was removed after four months. The patient has been followed for six years. She is able to walk with braces and perform most activities independently. She is progressing well with fine motor and self care skills. She does have neurogenic bowel and bladder. By choice from her parents, her bladder is managed with incontinence into a diaper. VCUG has revealed no vesicoureteral reflux. Recent urodynamics did show some leakage with filling but no abnormal detrusor contractions or pathologic bladder pressures. She has had no recurrence of prolapse, urinary obstruction or hydronephrosis (Fig.2). 

**Figure:**
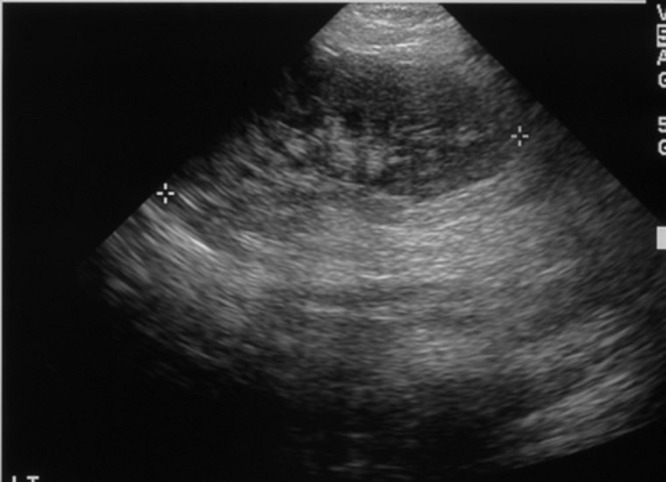
Figure 2: Renal ultrasound approximately one year following reduction of prolapse, showing complete resolution of left hydronephrosis. Again, right hydronephrosis had also resolved.

## DISCUSSION

Neonatal genital prolapse is a rare condition manifesting at birth or within the first few days of life and is most commonly associated with spinal cord malformations (82-86%) [1]. Defects of innervation to the pelvic floor musculature and ligamentous support can cause a flaccid paralysis, allowing downward protrusion of the abdominal and pelvic organs. The remaining cases of neonatal genital prolapse occur with a presumably normal suspensory system damaged by birth trauma, abnormalities of the cervix, or prolonged birth, especially in the breach position [2]. In 1955, Malpas separated these two etiologies into primary and secondary causes of neonatal genital prolapse, respectively [3]. Our patient would be classified as a case of primary genital prolapse with a neurologic etiology and additional resultant findings of neurogenic bowel and bladder.

Although genital prolapse was first documented in the Egyptian medical papyrus, the Ebers papyrus, dated 1550 BC, neonatal prolapse was not reported until 1723. Relatively few cases have been documented in modern literature. Diagnosis, however, is often unmistakable on exam with a characteristic fleshy mass projecting from the vagina. The extent of prolapse may vary, ranging from isolated vaginal prolapse to include the cervix or uterine corpus as well [2]. Differential diagnoses include vaginal polyps, urethral prolapse, paraurethral cysts, and rhabdomyosarcoma. Diagnosis can be readily confirmed with restoration of normal anatomy upon reduction of the prolapse and recognition of associated spinal cord abnormalities [3].

Multiple treatment modalities have been described, and conservative treatments are largely curative. The most conservative approach, single or repeated digital reduction of the prolapsed organs, has met with varied results. In 1927, Noyes treated prolapse in a child with spina bifida with digital reduction, but this method was unsuccessful. The infant died within a few days of life from meningitis [2]. However, Fraser (1961) and more recently, Bataypour (1992), reported successful treatment with digital reduction in children with no spinal cord abnormalities [1,4]. As genital edema resolves and the withdrawal of maternal estrogens takes place, the pelvic viscera are likely to maintain their proper position, especially in children with no abnormalities of sacral innervation. It seems that in these otherwise normal neonates, manual reduction, and at most, pessary placement, is sufficient to cure genital prolapse. The first report of a pessary as treatment for prolapse in neonates was by Dixon et al in 1974. The pessary was created from a 1-inch rolled Penrose drain with a 1-0 silk guy string attached to facilitate removal. Treatment was successful but was associated with vaginitis [3]. The pessary technique has been modified with different materials, with or without the addition of estrogen or antibiotic creams. There have been several reports of success with this management, even in children with spinal cord abnormalities [4]. Successful reduction with a bottle nipple or foley catheter with partially inflated balloon have also been reported [3,5].

More radical approaches to prolapse in neonates with congenital spinal cord anomalies have been described in the literature when conservative treatment had failed. This is indicated with recurrent prolapse despite repeated reduction or when there is evidence of vaginal mucosal hypertrophy or ulceration. Many fixation techniques have been described as effective and safe, including uterine ventrosuspension, sling, sacral cervicopexy, or abdominal sacrocolpopexy [6,7]. Ajabor and Okojie (1976) used hypertonic saline packs to reduce edema from prolapse and then partially fused the labia majora for two weeks with total resolution [8]. Hysterectomy or amputation of the cervix have been suggested but are rarely, if ever, necessary. This is in stark contrast from the adult population in which pelvic organ prolapse (usually secondary to a combination of vaginal childbirth, hypoestrogenism, and obesity) is extremely common but generally requires obliterative or reconstructive surgery for cure. Prolapse reduction and pessary placement in adult women are utilized to improve symptoms, reduce progression and delay surgical treatment [9]. In neonates, prompt and definitive treatment of genital prolapse is advocated to preserve fertility and to allow for more morbid conditions associated with spinal cord abnormality, such as hydrocephalus, to take precedence [2]. The choice of treatment in our patient led to swift and definitive resolution of her prolapse and potentially curbed recurrent urinary tract infection and future renal damage. In retrospect, ureteral stenting was not necessary as the cause of her obstruction was not related to a ureteral anomaly. However, due to concern for megaureter, this was thought to be the safest method of treatment at the time.

To our knowledge, this condition has never been reported in association with urinary obstruction and hydronephrosis. In the adult population, prevalence of hydronephrosis is estimated to be between 7.7 % and 34.6% and is generally believed to correlate with the degree of prolapse [10]. In this patient with development of grade IV hydronephrosis within a short time period, and with a known cause of obstruction, definitive treatment was deemed necessary before deterioration of renal function ensued. This patient experienced complete resolution of her prolapse as well as her hydronephrosis with relatively conservative digital reduction and transient placement of a vaginal pessary. This case illustrates a rare condition in the neonatal population associated with a potentially morbid urologic sequela. However, with prompt diagnosis and conservative treatment by manual reduction and vaginal pessary placement, cure for both conditions is attainable.

## Footnotes

**Source of Support:** Nil

**Conflict of Interest:** None

